# Photo-Induced Birefringence in Layered Composite Materials Based on Ge–Te–In and Azo Polymer Prepared Through Different Methods

**DOI:** 10.3390/ma18163837

**Published:** 2025-08-15

**Authors:** Yordanka Trifonova, Ani Stoilova, Deyan Dimov, Georgi Mateev, Vladislava Ivanova, Iliyan Mitov, Olya Surleva

**Affiliations:** 1 Department of Physics, University of Chemical Technology and Metallurgy, 8 Kl. Ohridski Blvd., 1756 Sofia, Bulgaria; ani_stoilova@uctm.edu (A.S.); ivanova_vl@uctm.edu (V.I.);; 2 Institute of Optical Materials and Technologies, Bulgarian Academy of Sciences, Acad. Georgi Bonchev Bl. 109, 1113 Sofia, Bulgaria; 3 Department of Metallurgy, University of Chemical Technology and Metallurgy, 8 Kl. Ohridski Blvd., 1756 Sofia, Bulgaria

**Keywords:** layered composite materials, birefringence, indium containing chalcogenides, azo polymers

## Abstract

Bulk chalcogenides from the system (GeTe_4_)_1−x_In_x_, where x = 0; 5 and 10 mol%, were synthesized by a two-step melt quenching technique. New layered composite materials based on them and the azo polymer [1-4-(3-carboxy-4-hydrophenylazo) benzensulfonamido]-1,2-ethanediyl, sodium salt] has been prepared through spin coating, electrospray deposition and via vacuum-thermal evaporation of the chalcogenide and spin coating of the azo polymer onto it. Using the latter technology, a material consisting of one chalcogenide and one azo polymer film and three chalcogenide and three azo polymer films has been fabricated. The carried-out SEM analysis shows that in the materials, initially prepared as a bilayer and multilayer structure, diffusion at the chalcogenide/polymer interface occurs leading to the formation of a homogenous composite environment. Birefringence was induced at 444 nm in all the fabricated thin film materials. The highest value of the maximal induced birefringence has been measured for the material fabricated as a stack, Δn_max_ = 0.118. For the material prepared as a bilayer structure and the composite material obtained through electrospray deposition, the maximal induced birefringence takes values of Δn_max_ = 0.101 and Δn_max_ = 0.095, respectively. The sample prepared via spin coating of the chalcogenide/PAZO dispersion has the lowest value of the maximal induced birefringence (Δn_max_ = 0.066) in comparison to the pure PAZO polymer film (Δn_max_ = 0.083).

## 1. Introduction

Azobenzene-containing polymers and chalcogenides represent two distinct classes of photo-responsive materials, each with unique properties and applicability. The first one consists of organic materials, which, when illuminated with linearly polarized light, exhibit optical anisotropy as a result of trans–cis photoisomerization. Based on that, they are intensively studied for use in reversible optical data storages, photonic devices, drug delivery systems, etc. [1,2,3]. Chalcogenides, on the other hand, are inorganic materials, highly valued for their ability to undergo reversible phase changes between amorphous and crystalline states using heat generated by electrical or light stimuli. Rewritable DVDs based on Ge_2_Sb_2_Te_5_ and AgInSbTe have been successfully commercialized and are widely used today [4,5]. Structurally, the azobenzene-containing polymers are more flexible than the chalcogenides; on the other hand, the latter offer more mechanical and thermal stability. Optically, the azo polymers have a typically lower refractive index and exhibit lower nonlinearity than the chalcogenides. Most of them are transparent in the UV to visible region, where the chalcogenides absorb. Regarding the technology of thin films fabrication, azobenzene-containing polymer films are usually prepared using solution-based techniques as chalcogenides often require high temperature and vacuum environments. Combining chalcogenides with azobenzene-containing polymers in composite materials offers the potential for development of advanced multifunctional systems. However, few scientific studies focus on this topic [6,7,8,9] which may be due to the different mechanisms by which the azo polymers and the chalcogenides respond to light irradiation [2,10]. Despite the differences, there are many similarities between them. For instance, upon irradiation with polarized light, many chalcogenides also become optically anisotropic; marked volume changes, fluidity and mechanical motions commonly accompany the photo-structural behavior of these two classes of materials [10].

In this study, we present the synthesis of chalcogenides from the system Ge–Te–In and the preparation of layered composite materials based on them and the azo polymer [1-4-(3-carboxy-4-hydrophenylazo) benzensulfonamido]-1,2-ethanediyl, sodium salt], abbreviated below as PAZO. The synthesis of chalcogenides from the same system and with the same composition has already been reported [11]. The difference in this investigation is that the alloy was left in the furnace to cool slowly down to room temperature. The choice of chalcogenides containing Ge–Te arises from previously reported high degrees of Ge and Te interdiffusion in Sb_x_Te_1−x_/Ge bilayer films [12]. We used indium as the dopant instead of antimony, as it has a lower toxicity [13] and integrates more homogeneously into the GeTe lattice compared to Sb [14]. The azo polymer PAZO is commercially available, absorbs in the visible range where the used chalcogenide also absorbs and has been widely used as photo-anisotropic polymer matrix in composite materials doped with various sized, shaped and nature particles, developed for optoelectronic application [15]. Birefringence has been induced in the new layered materials and the influence of the preparation technique on the optical performance has been discussed. To the best of our knowledge, no results from measuring the photo-induced birefringence in layered composite materials based on Ge–Te–In and the azo polymer PAZO have been reported.

## 2. Materials and Methods

The bulk samples from the system (GeTe_4_)_1−x_In_x_, where x = 0; 5 and 10 mol%, were synthesized by a two-step melt quenching technique. Pure Ge, Te and In were used as elemental precursors. They were mixed in appropriate stoichiometric ratios, sealed in a quartz ampoule under vacuum (1.33 × 10^−3^ Pa) and heated in a furnace (Beta-électriqueVecon 10, VECTOR 1 Ltd., Plovdiv, Bulgaria) at a constant rate of 5 × 10^−2^ K/s up to 750 °C, kept at this temperature for one hour and afterwards heated up to 1100 °C and kept at this temperature for 2 h. The furnace was then turned off, and the molten mixture was left in it to cool slowly down to room temperature. Finally, the bulk samples were taken from the quartz tube and cut into slices.

The azo polymer poly [1-4-(3-carboxy-4-hydrophenylazo) benzensulfonamido]-1,2-ethanediyl, sodium salt], denoted as PAZO throughout the text below, was purchased from Sigma Aldrich (St. Louis, MO, USA, Prod. #346411).

For the preparation of the non-doped PAZO polymer film, 75 mg from the PAZO polymer were dissolved in 2000 µL methanol by means of a magnetic stirrer (IKA^®^ RET B 8000, IKA Ltd., Wilmington, DE, USA). Afterwards, the mixture was stirred for one hour at 1700 rpm at room temperature. A drop of 160 µL from the resulting solution was deposited on a quartz substrate and spin coated for 60 s at 1000 rpm.

For the preparation of the composite film doped with Ge_18_Te_72_In_10_ particles through spin coating, the synthesized bulk sample was mechanically grinded and sieved through openings of 800 nm. A total of 18 mg from the particles were than dispersed in 3 mL methanol through sonication at room temperature for 1 h (Elmasonic P 60 H, frequency 37 kHz, Elma Schmidbauer GmbH, Singen, Germany). Then, 18.7 mg from the azo polymer solution prepared for the fabrication of the pure PAZO polymer and 0.39 mg from the particle’s dispersion were mixed and sonicated at room temperature for 30 min, so that a dispersion containing 2 wt.% Ge_18_Te_72_In_10_ in PAZO was prepared. A drop of 160 µL from the resulting dispersion was deposited on a quartz substrate and spin coated for 60 s at 1000 rpm.

For the preparation of the composite film doped with Ge_19_Te_76_In_5_ particles through electrospray deposition, 2 mL from the same dispersion was used for the spin coating. The flow rate was 2 mL/h, the deposition time was about 30 min and the applied voltage 16 kV. It used a purpose-built electrospray system consisting of a stainless-steel capillary nozzle connected to a high voltage generator switched to positive polarity. The distance between the nozzle tip and the table was 6 cm.

For the preparation of the bilayer structure and the stacked material, the bulk chalcogenide was evaporated from a quartz evaporator on a quartz substrate using the vacuum-thermal evaporation technique. The layer was deposited at evaporation rate of 1 Å/s. The pressure was kept below 2 × 10^−6^ mbar. The thickness and the deposition rate were controlled by a QCM-quartz crystal microbalance (INFICON SQM-160, Inficon Ltd., Bad Ragaz, Switzerland). The azo polymer film was deposited onto the chalcogenide film by spin coating 150 µL from the solution prepared for the fabrication of the non-doped PAZO polymer film for 60 s at 1000 rpm.

For clarity, the techniques for the preparation of the samples and the different methods for the layered composite materials, as discussed in the text, are listed in Table 1.

The XRD experiments were carried out at “Philips” powder diffractometer working in the Bragg–Brentano (θ–2θ) geometry, with a CuKα radiation (λ = 1.54060 × 10^−10^ m) and a graphite monochromator for the reflected beams. The XRD patterns were obtained after 75 s at a constant scan rate and reflection angle of 2θ in the scan range from 15° to 70° with a scan step of 0.05°.

A scanning electron microscope (EVO MA 10, Carl Zeiss GmbH, Oberkochen, Germany) connected with an EDX (Energy-Dispersive X-ray detector system, Bruker, Billerica, MA, USA) and a Philips 515 scanning electron microscope (Philips Electron Optics, Eindhoven, The Netherlands) were used to study the surface morphology of the layered composites and the chemical composition of the Ge_18_Te_72_In_10_ thin film.

The AFM study was performed using an atomic force microscopy Bruker CP-II and MFP-3D, Asylum Research, Oxford Instruments, Billerica, MA, USA.

The thicknesses of the films were determined by a high precision profilometer “Talystep”, Taylor Hobson, San Diego, CA, USA, with an accuracy of ±5 nm. The measured thickness for sample 1 was about 200 nm, for sample 2–170 nm, for sample 3–387 nm, for sample 4–170 nm and for sample 5–500 nm. The thickness of the vacuum-evaporated chalcogenide film was about 20 nm.

The density of the bulk samples was estimated by the pycnometer technique, with an accuracy of ±0.7%, using water as the immersion fluid. The compactness (δ), the molar, volume $\left(V_{m}\right)$ and the free volume percentage (FVP) of the materials were calculated according to Equations (1)–(3), respectively, where *ρ* is the density of the sample, *ρ_i_*, *A_i_* and *c_i_* are the density, the atomic mass and atomic fraction of the i-th component and *V_T_* is the theoretical molar volume determined as a sum of the molar volumes of the components multiplied by the atomic fraction of each of them.

(1) $$\delta =\frac{\sum_{i}\frac{c_{i}A_{i}}{\rho_{i}}-\sum_{i}\frac{c_{i}A_{i}}{\rho }}{\sum_{i}\frac{c_{i}A_{i}}{\rho }}$$

(2) $$V_{m}=\frac{1}{\rho }\sum_{i}c_{i}A_{i}$$

(3) $$FVP=\frac{V_{m}-V_{T}}{V_{m}}\times 100,\%$$

To induce birefringence, it was used a vertically polarized pump laser beam (Coherent cube, intensity 330 mW/cm^2^) emitting at 444 nm, and as probe beam a linearly polarized at 45° beam from a diode-pumped solid-state (DPSS) laser, with wavelength 635 nm. The value of the induced birefringence was calculated according Equation (4):

(4) $$\Delta n=\frac{\lambda \delta }{2\pi d}$$

where *λ* is the wavelength of the probe laser, *d* is the film thickness and S_2_ and S_3_ are two of the four components of the Stokes vector.

## 3. Results

### 3.1. XRD and EDS Analysis

**Figure 1:**
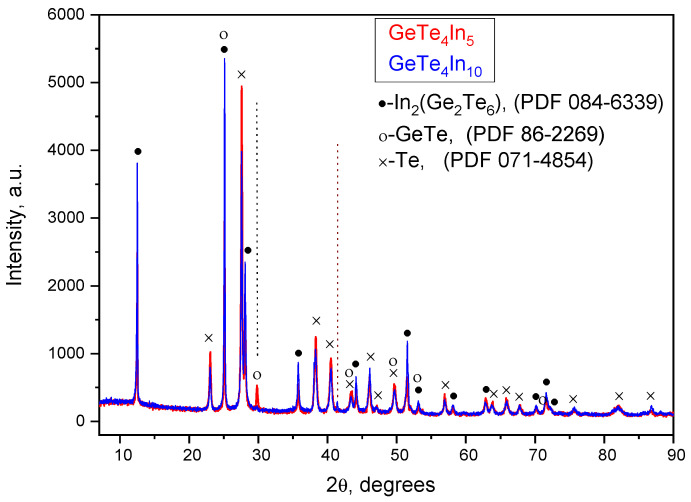
XRD patterns of sample Ge_19_Te_76_In_5_ and Ge_18_Te_72_In_10_.

The X-ray diffraction study showed that the synthesized new bulk materials are in crystalline form (Figure 1). The following three crystal phases were identified: hexagonal Te (PDF No 071-4854), rhombohedral GeTe (PDF No 86-2269) and rhombohedral In_2_(Ge_2_Te_6_) (PDF No 084-6339).

The hexagonal phase of tellurium consists of infinite zigzag chains of Te atoms. These chains are held together by weak interactions, which contribute to the thermodynamic stability of the phase under certain conditions. The presence of free Te atoms can be attributed to the tellurium enrichment in the studied materials, which facilitates the formation of isolated Te chains within the structure. In the rhombohedral GeTe phase, each germanium atom is bonded to six equivalent tellurium atoms, forming a mixture of corner- and edge-sharing GeTe_6_ octahedra. Conversely, each Te atom is bonded to six equivalent Ge atoms, creating a similar mixture of corner- and edge-sharing TeGe_6_ octahedra. In chalcogenide materials such as GeTe, atoms preferentially form bonds with atoms of a different type rather than with those of the same type. This tendency promotes the formation of heteropolar bonds, such as Ge–Te, Ge–In and Te–In in the synthesized new materials. For the sample containing 10 mol% In, the intensity of the XRD peaks corresponding to the GeTe phase is markedly reduced. This decrease is attributed to the partial substitution of Ge and Te atoms by In, which lowers the overall concentration of GeTe units in the crystal structure. The rhombohedral In_2_(Ge_2_Te_6_) phase exhibits a layered, two-dimensional crystal structure consisting of three stacked InGeTe_3_ planes per unit cell, in which each Ge atom is bonded to six equivalent Te atoms, forming GeTe_6_ octahedra that share edges. The Te atoms are bonded in a distorted trigonal non-coplanar geometry with two equivalent Ge atoms and one In atom. Each In atom is bonded in a trigonal planar geometry with three equivalent Te atoms. As the In content in the samples increases, the intensity of the XRD peaks associated with this phase becomes more pronounced, indicating an increased concentration in the In_2_(Ge_2_Te_6_) phase in the materials.

**Figure 2:**
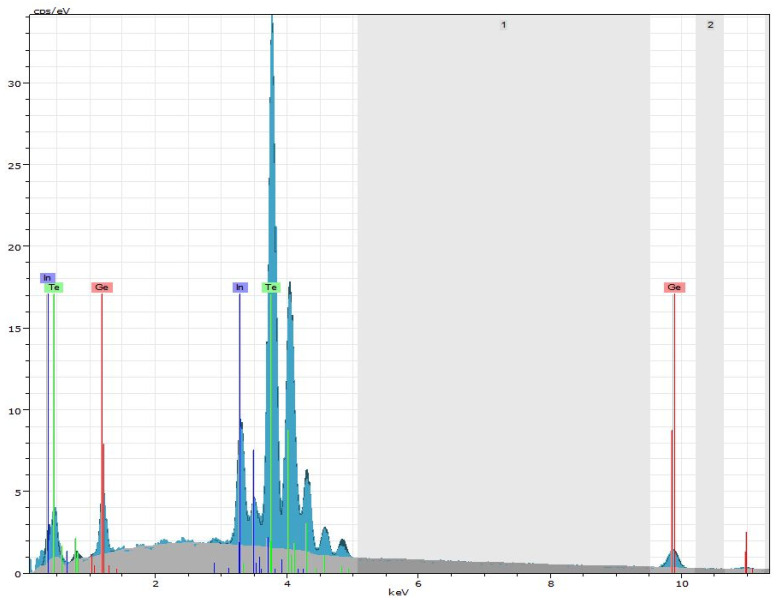
EDS spectrum of Ge_18_Te_72_In_10_ thin film.

For the preparation of the layered composites, chalcogenide with composition Ge_18_Te_72_In_10_ was used. Figure 2 shows the EDS spectrum of Ge_18_Te_72_In_10_ thin film. The obtained results from the EDS analysis are summarized in Table 2, which indicate the presence of Ge, Te and In in concentrations in direct correspondence with those theoretically determined.

### 3.2. AFM Analysis

**Figure 3:**
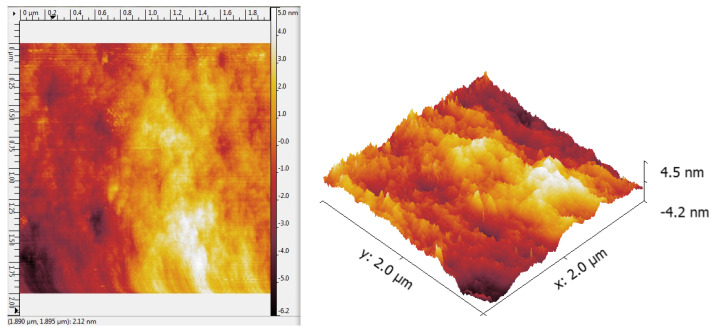
AFM image of a (GeTe_4_)_90_In_10_ layer.

**Figure 4:**
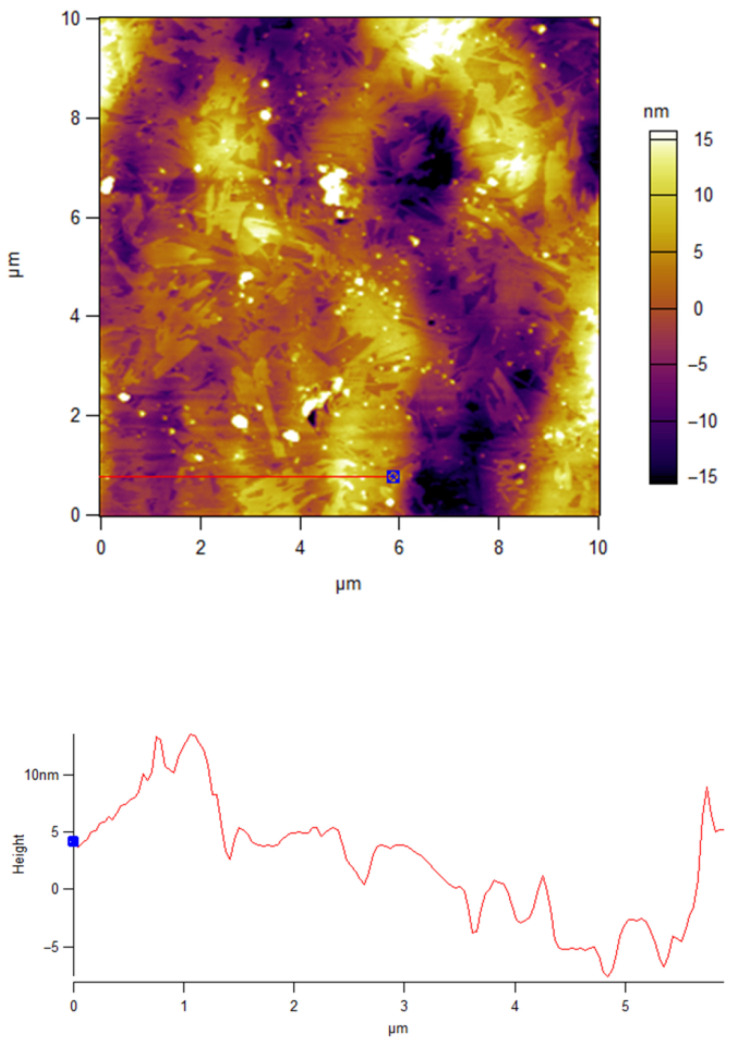
AFM image and surface roughness profile of the PAZO polymer layer.

Figure 3 and Figure 4 present AFM images of the Ge_18_Te_72_In_10_ and the PAZO polymer layer prepared through the same methods under the same conditions as used for the fabrication of the bilayer structure and the stack. A high-quality silicon wafer with an area of 1 × 10^−4^ m^2^ was used. The thickness of the prepared layers is about 370 ± 4.5 nm for the chalcogenide film and 1750 ± 15 nm for the azo polymer layer.

As seen from the AFM images, both the Ge_18_Te_72_In_10_ chalcogenide and the azo polymer layer exhibit low surface roughness.

### 3.3. SEM Analysis

**Figure 5:**
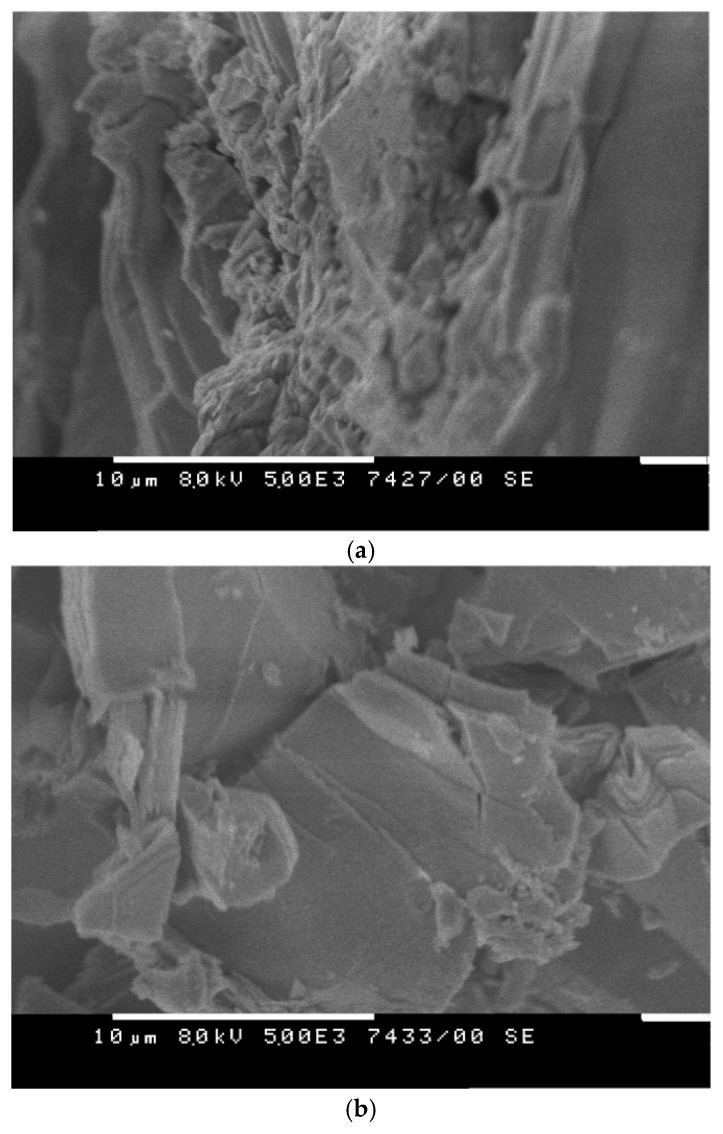
SEM images of bulk Ge_19_Te_76_In_5_ (**a**) and Ge_18_Te_72_In_10_ (**b**).

The micrograph of the sample doped with 5 mol% indium, Figure 5a, reveals the formation of triangular, rock-like structures corresponding to the tellurium (Te) phase. Laminar-like features with numerous edges and steps are also visible, consistent with the known morphology of the Ge–Te and In_2_(Ge_2_Te_6_) phases. The SEM image of the sample doped with 10 mol% indium, Figure 5b, exhibits a more planar, layered morphology, reflecting the increased indium content and the higher concentration of the In_2_(Ge_2_Te_6_) phase.

**Figure 6:**
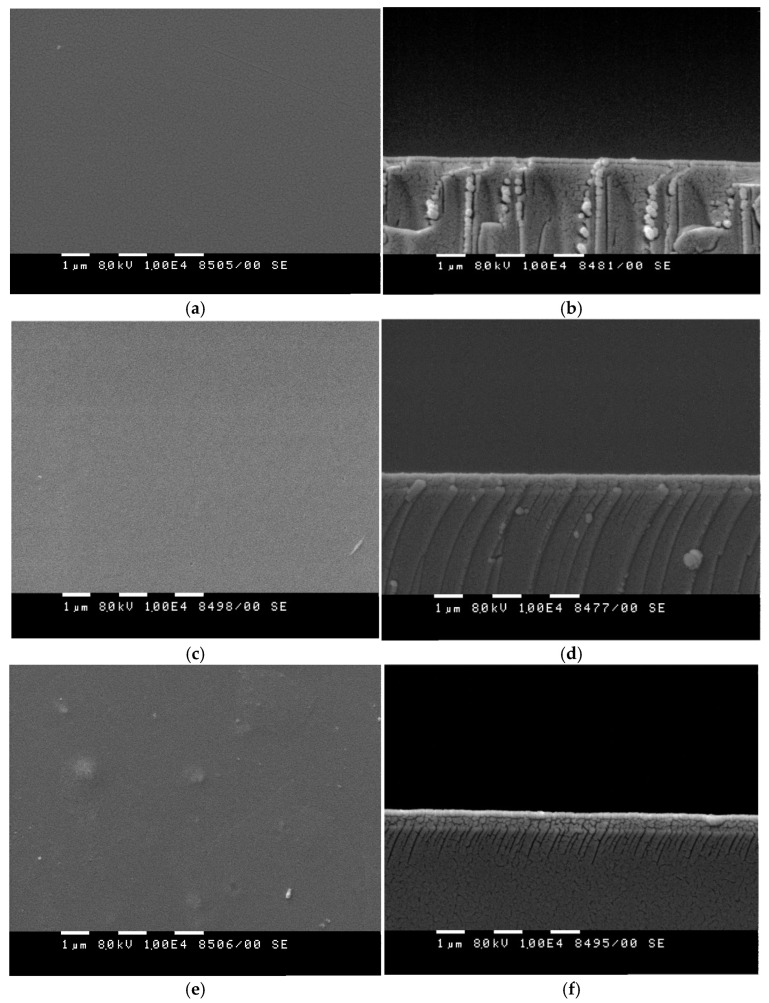
SEM images from the surface and cross-section of sample 4 (**a**,**b**), sample 5 (**c**,**d**) and of the sample prepared through spin coating the mixture containing Ge_18_Te_72_In_10_ dispersed in the PAZO polymer (**e**,**f**).

Figure 6 presents SEM images from both top-view and cross-sectional perspectives of sample 4 (a, b), sample 5 (c, d) and sample 3 (e, f). The surface micrographs of all the studied samples reveal the presence of crystallites with planar, polygon-like shapes. Notably, sample 4 exhibits larger crystallites compared to sample 5. On the surface of sample 3, some clusters are to observed. At the used SEM magnification, the bilayer and stacked films present a visually smooth surface, as shown in Figure 6a,c, which is expected considering the results obtained from the AFM analyses carried out on a single chalcogenide film and an azo polymer layer. Moreover, the azo polymer layer being substantially thicker in the prepared bilayer structure and stack than the chalcogenide film and spin coated from concentrated polymer solutions at low spin speeds, favors planarization of the underlying nanoscale topography [16]. The cross-sectional image of sample 4, initially prepared as a bilayer structure, shows a lack of distinct layers. A well-defined composite layer with thickness of about 170 nm, which remains uniform along the entire length of the substrate is also observed. Similarly, the cross-section of sample 5, deposited as a stack, shows the formation of a composite layer, approximately 500 nm thick, exhibiting a homogeneous structure throughout its depth. Within this layer, tightly packed polyhedral-like structures are observed, consistent with the polycrystalline morphology seen on the surface. The SEM analysis of samples 4 and 5 indicates interdiffusion at the chalcogenide/polymer interface, leading to the formation of a single, homogeneous and continuous composite layer. The cross-sectional image of the sample prepared by spin coating the Ge_18_Te_72_In_10_/PAZO mixture shows at a well-defined composite layer with a uniform thickness of approximately 170 nm and a homogeneous structure at the used magnification. The chalcogenide particles appear evenly dispersed throughout the polymer matrix.

### 3.4. Physico-Chemical Properties

Table 3 presents the obtained values for the density, compactness, molar volume and free volume percentage of all the samples studied. The incorporation of 5 mol% indium into the Ge–Te matrix results in a more densely packed structure. For the sample doped with 10 mol% indium, the value of the free volume percentage (FVP) is higher than for Ge_19_Te_76_In_5_. Structural investigation on In_2_Ge_2_Te_6_ crystals has shown that single indium atoms occupy interstitial states in the Ge–Te host lattice [17,18]. It could be assumed that the less flexible structure (higher compactness and lower FVP) of the sample containing 5 mol% In compared to the one doped with 10 mol% is due to the incorporation of the indium atoms within the existing Ge–Te network. At 10 mol% In content, indium starts to disrupt the Ge–Te bonding network leading to increased free volume and structural flexibility.

### 3.5. Measuring the Photo-Induced at 444 nm Birefringence

**Figure 7:**
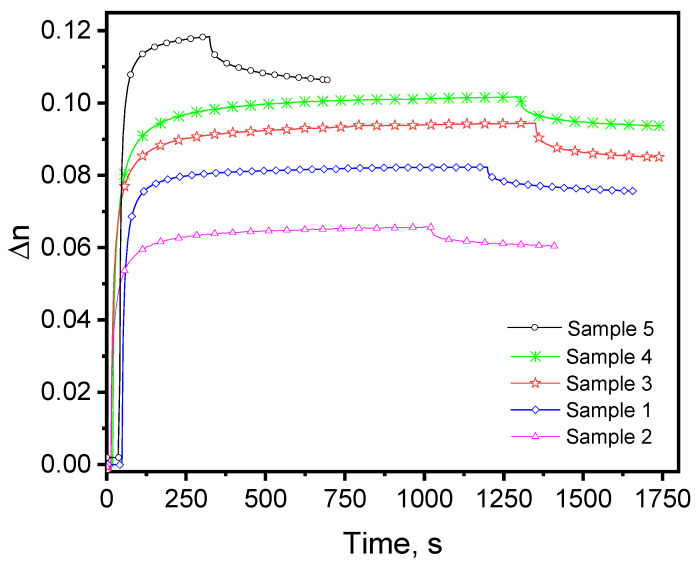
Birefringence kinetics for the samples studied induced at 444 nm.

Figure 7 presents the results from measuring the kinetics of birefringence induced at 444 nm in the fabricated thin film materials containing the azo polymer PAZO and the Ge_18_Te_72_In_10_ chalcogenide.

The highest value of the maximal induced birefringence was the stacked material, Δn_max_ = 0.118. For the bilayer structure and the composite material obtained through electrospray deposition, the maximal induced birefringence has values of Δn_max_ = 0.101 and Δn_max_ = 0.095, respectively. The sample prepared via spin coating has the lowest value of the maximal induced birefringence (Δn_max_ = 0.066) in comparison to the pure PAZO polymer film (Δn_max_ = 0.083).

## 4. Discussion

The lowest value of the maximal induced birefringence for the composite material prepared by dispersing the chalcogenide particles into the azopolymer solution and then spin coating the mixture can be explained with the large size of the Ge_18_Te_72_In_10_ particles obtained through grinding and saving of the bulk material. Studies have shown that incorporating particles larger than a certain size into PAZO leads to lower Δn_max_ compared to the non-doped azo polymer film [19,20,21]. The decrease has been attributed to increased scattering and a reduced surface-to-volume ratio. Larger particles aggregate more easily than smaller ones forming clusters that further intensify the above-mentioned effects. In the electrospray technique, the applied high-voltage electric field promotes more uniform particle distribution within the film as Coulombic repulsion between similarly charged droplets prevents aggregation. As the SEM image of the composite material prepared through spin coating of the PAZO/Ge_18_Te_72_In_10_ dispersion reveals some clumps on the surface, we attribute the higher value of the Δn_max_ measured for the sample prepared from the same dispersion but through electrospraying to the above-mentioned advantages of the electrospray technology. The SEM cross-sections of the materials initially prepared as bilayer and multilayer structures show an absence of distinct layers. At the used magnification, the SEM images reveal the formation of a composite environment that might facilitate the photo-induced birefringence through several mechanisms including nanoparticle-like effects of the chalcogenide inclusions; dipole–dipole interplay between the PAZO and the chalcogenide; and the creation of strong local refractive index contrast. Chalcogenide inclusions can behave like embedded nanoparticles in the polymer matrix—creating extra free volume that boosts azo-chromophore mobility or—acting as scattering centers that activate “off-plane” chromophores. Ge–Te chalcogenides, particularly when prepared as thin films or at interfaces, exhibit surface dipoles arising from broken inversion symmetry [22]. Dipole–dipole interactions between the PAZO and the chalcogenide can prevent close antiparallel packing of the azo-chromophores leading to a more effective alignment of the polymer chains. A composite environment based on materials with significantly different refractive indices creates localized anisotropic regions producing a larger net Δn than the polymer alone. To convert refractive index contrast into net birefringence, sub-wavelength-sized structures are required, which has not been achieved by the spin coated and electrospray deposited materials. The sample prepared through evaporation of the three layers of the chalcogenide separated by a spin coated azopolymer film shows more mixed, less stratified topology compared to the material fabricated through evaporation of one Ge–Te–In layer and spin coated on a PAZO polymer film. In the chalcogenide/polymer stack, the interfaces between the successive layers provide additional sites for interdiffusion. Each chalcogenide layer can diffuse into the adjacent polymer layer, and vice versa, leading to a more homogeneous interphase. This increased interdiffusion results in a more mixed and less stratified topology, which reduces the overall structural rigidity of the material facilitating greater chromophore mobility and therefore a higher Δn_max_.

## Figures and Tables

**Table 1:** Samples preparation techniques.

Preparation Technique	Sample
Spin coating of PAZO dissolved in methanol, pure PAZO polymer film	Sample 1
Spin coating of mixture containing PAZO polymer dissolved in methanol and Ge_18_Te_72_In_10_ particles dispersed in methanol	Sample 2
Electrospray deposition of mixture containing PAZO polymer dissolved in methanol and Ge_18_Te_72_In_10_ particles dispersed in methanol	Sample 3
Vacuum-thermal evaporation of one chalcogenide layer and spin coating an azo polymer layer onto it	Sample 4
Vacuum-thermal evaporation of three chalcogenide layers separated by one spin coated azopolymer film	Sample 5

**Table 2:** EDS elemental analysis of thin Ge_18_Te_72_In_10_ film prepared through vacuum-thermal evaporation under the same conditions used for the fabrication of the composites.

Element	Series	Unn., wt.%	C norm., wt.%	C Atom., at.%	C Error, %
Germanium	K-series	10.92	11.16	17.86	0.4
Tellurium	L-series	75.17	76.83	69.98	2.2
Indium	L-series	11.75	12.01	12.16	0.4
	Total	97.84	100.00	100.00	

**Table 3:** Physico-chemical properties of the bulk samples studied.

Sample	Density, 10^3^ kg/m^3^	Compactness, 10^−2^	Molar Volume, 10^−5^ m^3^/mol	FVP, %
Ge_20_Te_80_	5.560	−8.9	2.10	-
Ge_19_Te_76_In_5_	5.926	−3.8	1.97	3.77
Ge_18_Te_72_In_10_	5.533	−10.9	1.10	10.09

## Data Availability

The original contributions presented in this study are included in the article. Further inquiries can be directed to the corresponding author.
